# Unbiased data-driven analysis of five amyloid-beta peptides for biomarker investigations in familial Alzheimer’s disease

**DOI:** 10.1093/braincomms/fcag105

**Published:** 2026-03-22

**Authors:** Isaac Llorente-Saguer, Rebecca Gabriele, Teisha Y Bradshaw, Claire A Leckey, Christopher R S Belder, Lucía Chávez-Gutiérrez, Rohan de Silva, Nick C Fox, Selina Wray, Neil P Oxtoby, Charles Arber

**Affiliations:** Department of Medical Physics and Biomedical Engineering and UCL Hawkes Institute, University College London, London WC1V 6LJ, UK; Department of Neurodegenerative Disease, UCL Queen Square Institute of Neurology, London WC1N 3BG, UK; Department of Clinical and Movement Neurosciences, Reta Lila Weston Institute, UCL Queen Square Institute of Neurology, London WC1N 3BG, UK; Translational Mass Spectrometry Research Group, UCL Great Ormond Street Hospital Institute of Child Health, University College London, London WC1N 1EH, UK; Dementia Research Centre, UCL Queen Square Institute of Neurology, London WC1N 3BG, UK; UK Dementia Research Institute at UCL, London WC1E 6BT, UK; Dementia Research Centre, UCL Queen Square Institute of Neurology, London WC1N 3BG, UK; Adelaide Medical School, The University of Adelaide, Adelaide, SA 5000, Australia; Department of Neurology, Royal Adelaide Hospital and Queen Elizabeth Hospital, Adelaide, South Australia 5005, Australia; VIB-KU Leuven Center for Brain & Disease Research, Leuven 3001, Belgium; Department of Neurosciences, Leuven Research Institute for Neuroscience and Disease (LIND), KU Leuven, Leuven 3001, Belgium; Department of Clinical and Movement Neurosciences, Reta Lila Weston Institute, UCL Queen Square Institute of Neurology, London WC1N 3BG, UK; Department of Neurodegenerative Disease, UCL Queen Square Institute of Neurology, London WC1N 3BG, UK; Dementia Research Centre, UCL Queen Square Institute of Neurology, London WC1N 3BG, UK; UK Dementia Research Institute at UCL, London WC1E 6BT, UK; Department of Neurodegenerative Disease, UCL Queen Square Institute of Neurology, London WC1N 3BG, UK; Department of Computer Science and UCL Hawkes Institute, University College London, London WC1V 6LJ, UK; Department of Neurodegenerative Disease, UCL Queen Square Institute of Neurology, London WC1N 3BG, UK

**Keywords:** amyloid, data-driven, biomarker, iPSC, Alzheimer’s disease

## Abstract

Changes to the relative abundance of amyloid-beta (Aβ) peptides are hallmarks of Alzheimer’s disease. Induced pluripotent stem cell (iPSC)-derived neurons offer a physiological model of Aβ production. We employed unbiased, data-driven analyses to investigate combinations of Aβ peptides as Alzheimer’s disease biomarkers and the relative contribution of peptides to Alzheimer’s disease pathogenesis.

We measured Aβ37, Aβ38, Aβ40, Aβ42 and Aβ43 in 10 iPSC-neuronal cultures from *PSEN1* mutation carriers. We combined these data with published cell model data and used linear weighted combinations to (i) distinguish Alzheimer’s disease from controls, and (ii) predict age-at-onset for *PSEN1* mutations. Data-driven approaches distinguished Aβ42 and Aβ43 from shorter peptides, providing unbiased evidence for a greater association of Aβ42 and Aβ43 to disease pathogenesis, compared with shorter peptides (Aβ37, Aβ38 and Aβ40). Weighted linear combinations of Aβ peptides outperform Aβ42/40 and provide insights into relative peptide contribution as biomarkers. A representative weighted composite value ratio (wCVR) derived from all data, balancing both disease classification and age-at-onset prediction, was (21⋅Aβ37+10⋅Aβ38+69⋅Aβ40)/  (94⋅Aβ42+6⋅Aβ43). This work suggests a practical non-parametric harmonization approach to employing Aβ ratios as biomarkers for Alzheimer’s disease, from multiple sites and assays. Building on this foundation, we applied a new model using weighted composite value ratios, which outperform existing biomarkers across all tasks. This underscores the value of integrating multiple peptides and assigning optimized weightings. The study confirms the association of Aβ42 and Aβ43 with Alzheimer’s disease pathogenesis in a data-driven manner. Peptide weights further provide mechanistic insights into the relative contribution of each peptide to disease, such as a greater contribution of Aβ37 compared to Aβ38. The algorithm used herein can be further refined to improve biomarkers for Alzheimer’s disease.

## Teaser

Llorente-Saguer *et al*. developed an amyloid-beta-37 ELISA (Enzyme-Linked Immunosorbent Assay) and quantified five amyloid-beta peptides in 10 *PSEN1*-mutant lines. They introduce a harmonization method for multi-site data synthesis and then develop a data-driven weighted peptide ratio that informs on peptide pathogenicity and demonstrates potential for improved biomarker studies and risk stratification in Alzheimer’s disease.

## Graphical Abstract

**Graphical Abstract:**
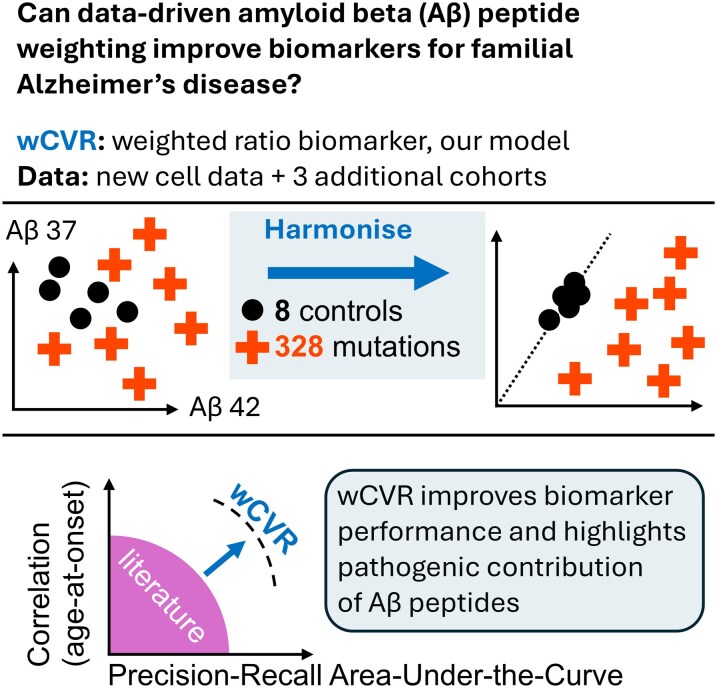

## Introduction

Mutations in *PSEN1* that cause early onset familial Alzheimer’s disease (fAD) reduce the processing of amyloid-beta (Aβ) from longer peptides (e.g. Aβ42 and Aβ43) to shorter peptides (e.g. Aβ37, Aβ38 and Aβ40).^1-3^ This causes a relative increase in longer, more aggregation-prone Aβ species, potentially predisposing Aβ aggregation, plaque pathology and disease.^4^ For this reason, a raised Aβ42/40 ratio is used in model systems to validate pathogenicity of fAD causing mutations,^5^ and is also supported by clinical data in plasma of mutation carriers^6^ (although, a recent study did not replicate these changes in plasma^7^). This phenomenon is distinct from the reduced Aβ42:40 ratio observed in plasma and CSF that is used to establish the presence of Aβ pathology in both fAD and sporadic Alzheimer’s disease (possibly due to higher sequestration of Aβ42 within plaques in the brain parenchyma).

Recently, Liu and colleagues reported that the Aβ37/42 ratio represents an improved biomarker for Alzheimer’s disease compared to Aβ42/40.^8^ In parallel work, a ratio of Aβ37 + 38 + 40/Aβ42 + 43 (termed ‘Aβshort/long’ hereafter) was shown to accurately predict the age-at-onset (AAO) of fAD mutations in cell models.^9,10^ Together, these studies show the value of incorporating a diversity of Aβ peptides into biomarker studies and prompt detailed exploration of peptide combinations as biomarkers.

*PSEN1* mutation carriers show remarkable heterogeneity in terms of age at onset,^11^ clinical symptoms^12^ and ratios of Aβ peptides.^10,13^ Indeed, certain *PSEN1* mutations significantly affect the Aβ43/40 ratio whilst leaving the Aβ42/40 ratio relatively spared.^13-16^ Differences in the relative abundance of Aβ peptides and clinical heterogeneity make it hard to interpret the relative importance of the different Aβ species, their contribution to disease mechanisms and their value as biomarkers.

Induced pluripotent stem cell (iPSC)-derived neurons offer a physiological human neuronal model of Aβ production that faithfully represents Aβ production *in vitro*^13,17,18^ (for review, see Arber *et al.*^19^). Importantly, iPSC-neurons also express one healthy *PSEN1* allele, in contrast to the cell models previously employed.^8-10^ Given the similarity of iPSC-neuronal Aβ profiles with blood plasma ratios from the same donor,^6^ coupled with the current interest in blood-based biomarker testing for Alzheimer’s disease, we sought to investigate Aβ ratios in our physiological model and employ unbiased data-driven techniques to probe their use for (i) distinguishing fAD from control samples and (ii) predicting AAO for fAD mutations.

## Materials and methods

### Cell culture

Details of all iPSC lines are presented in Supplementary Table 1. Fibroblast biopsies were collected from the upper arm of donors at the National Hospital for Neurology and Neurosurgery, with informed consent and Joint Research Ethics Committee approval (09/H0716/64). Individuals had confirmed mutations in *PSEN1* associated with familial Alzheimer’s disease, and where patients were symptomatic, clinical diagnosis was made at the National Hospital for Neurology and Neurosurgery or the UCL Dementia Research Centre. *PSEN1* E184D iPSCs were reprogrammed from fibroblasts for this study (Supplementary Fig. 1) using episomal reprogramming.^20^ Fibroblasts were collected with research ethics approval and informed consent (11/LO/0753). For the newly established E184D iPSC line, Sanger sequencing was performed (Source Bio) to demonstrate the presence of the mutation (Supplementary Fig. 1B), and a stable karyotype was confirmed using the Stem Cell Technologies hPSC Genetic Analysis kit (Supplementary Fig. 1C). Low-coverage whole-genome sequencing further confirmed a stable karyotype of the newly generated E184D patient-derived line (Supplementary Fig. 1D).^21^ The generation of other lines has been described in previous studies, and one well-characterized iPSC clone was employed per patient donor (Supplementary Table 1). iPSCs were grown in Essential 8 media on Geltrex substrate and passaged using EDTA mechanical passaging. In short, iPSCs were washed once with PBS before being exposed to 0.5 mM EDTA in PBS without magnesium and calcium. After 4 min, EDTA was removed, and iPSCs were washed off the plates as small cell colonies and passaged at a ratio of 1:6. iPSCs were fed daily with Essential 8 media and passaged every 5–6 days. iPSCs were differentiated into cortical neurons following published protocols^13-15,22^ using dual SMAD inhibition and extended neurogenesis. Triplicate data represents three independent neural inductions. In brief, iPSCs were pooled to become 100% confluent and then neural induction was performed for 10 days in N2B27 media with 10 μM SB431542 (Tocris) and 1 μM dorsomorphin (Tocris). Cells were passaged at Day 10 and Day 18 with dispase and cultured on laminin substrate, and cells were fed twice weekly from Day 10 onwards with fresh N2B27 media via half media changes. A final split using accutase was performed at Day 35 onto poly-ornithine- and laminin-coated plates. The final time point was taken as 100 DIV (days in vitro), where iPSC-neuronal media was conditioned for 48 h prior to Aβ analysis. Our previous work has shown that Aβ ratios are highly consistent between 100 and 200 DIV.^13^ All reagents were Thermo Fisher unless specified.^23^

### Aβ ELISAs

Aβ42, Aβ40 and Aβ38 were quantified using electrochemiluminescence (Meso Scale Discovery, 6E10 V-Plex). Aβ43 was quantified using the IBL Amyloid Beta (1–43) ELISA.

To develop a custom Aβ37 ELISA, we used the β-amyloid 1–37 (D2A6H) antibody from Cell Signaling Technology (carrier-free). Capture antibody was adsorbed to plates at 1 μg/ml (empirically optimized) using 0.05 M carbonate buffer (Sigma) for 24 h. Plates were washed and blocked with Starting Block (Thermo) before adding 50 μl of undiluted conditioned media or Aβ37 standards (ANASPEC, 0–500 pg/ml) in 20% SuperBlock (Thermo) together with 50 μl of capture antibody 6E10-HRP (Biolegend, 3 μg/ml [empirically optimized]). After washing, colour development was initiated with TMB (3,3′,5,5′-tetramethybenzidine) blue reagent and stopped using 2 M sulphuric acid. Absorbance was measured at 450 nm on a Tecan Spark 10 M plate reader.

Other cell data used in this study include published Aβ profiles generated by overexpression of fAD-linked *PSEN1* mutations in *PSEN1*/*PSEN2* double knockout cell lines; mouse embryonic fibroblast cell lines by Petit and colleagues,^9^ and by overexpression in HEK 293T cell lines by Liu and colleagues^8^ and Schultz and colleagues^10^ Table 1. These studies employed 6-plex electrochemiluminescence assays.

**Table 1:** Data description per cohort

Cohort	# Controls	# Cases	Method	(additional notes?)
iPSC	3	10	**Aβ38/40/42** MesoScale Discovery (6E10 V-Plex)	iPSC-derived neuronal conditioned media (100 DIV and 48-hour conditioning)
			**Aβ43** IBL Amyloid Beta (1–43) ELISA	
			**Aβ37** ELISA developed and described in this study	
Petit *et al*.^9^	1 (7)	26	**Aβ37/38/39/40/42/43**	*Psen1^−/−^Psen2^−/−^* MEFs.
			MesoScale Discovery (multi-spot electrochemilumiscent plates)	Retroviral transduction of PSEN1 and adenoviral transduction of APP_C99_. 24-hour conditioned media.
Liu *et al*.^8^	3	131	**Aβ37/38/39/40/42/43**	HEK *PS1*/*PS2* dKO cells.
			MesoScale Discovery (multi-spot electrochemilumiscent plates #L15XA-3)	Overexpression of PSEN1 and WT APP-C99. 24-hour conditioned media.
Schulz *et al*.^10^	1	161	**Aβ37/38/39/40/42/43**	HEK *PS1*/*PS2* dKO cells.
			MesoScale Discovery (multi-spot electrochemilumiscent plates #L15XA-3)	Overexpression of PSEN1 and WT APP-C99. 24-hour conditioned media.

For iPSC data, actual AAO for each donor was used where available; otherwise, AAO was predicted from individuals from within families with the same mutation as the donor.^12^ For Liu, Schultz and Petit, AAO was taken from the data within each publication.

Modelling was performed on the mean ratio for each individual patient-derived line or genotype.

## Modelling/analysis

### Data harmonization

Samples with any peptide measure equal to zero were removed (*N* = 14 from a total of 230; 3 from Petit *et al*.^9^ and 11 from Liu *et al*.^8^). Zeros could potentially be due to assay detection limits or errors, and impede the usage of the peptide as a denominator for these samples. Samples were averaged across batches to reduce the influence of measurement error. Data harmonization across all datasets was performed in two steps. First, to obtain relative concentrations, individual peptide intensities were normalized to the total peptide abundance within each sample. This step adjusts for differences in total sample loading. Second, to correct for assay-specific systematic biases, each peptide value was divided by the mean intensity of that specific peptide in the control samples of the respective dataset.

To evaluate biomarkers that include the addition of multiple peptides, each peptide measurement was scaled back to the relative control proportions based on data from Petit *et al*.^9^ Ratio biomarkers like the short/long^9^ involve a weighted sum where each peptide is weighted by its naturally occurring abundance in controls. This scaling step is essential because the relative abundances of Aβ peptides differ by orders of magnitude. Not scaling the data back will distort the setup for which the biomarker was designed.

## Statistical analysis

### Main metrics

We evaluate biomarkers in case–control classification and AAO regression scenarios. In classification experiments, we analyse the area under the receiver operator characteristic curve (ROC AUC), which plots sensitivity, or true positive rate TP/(TP + FN) against false positive rate FP/(FP + TN), where T/F indicates True/False and P/N indicates Positive/Negative. Since the data are strongly unbalanced (very few control samples), we also look at the precision–recall curve (PR AUC) of the smaller group, which plots control precision TN/(TN + FN) against control recall TN/(TN + FP), since PR AUC is a preferred metric in this scenario. To evaluate the correlation between the biomarker and the AAO, we employ the coefficient of determination (*R*^2^), using Pearson correlation, since peptide biomarkers have shown good linear correlation.^9^ Median and 95% confidence intervals (CIs) are reported for all metrics using 2000 bootstrapping samples, stratified in the classification case (to accommodate the heavy class imbalance).

### Peptide weighting analysis

We bootstrap the data-driven combinatorial biomarker ‘weighted composite value ratio’ (wCVR, explained later), running the search algorithm three times per bootstrap with random initializations, to investigate the effect of the weighting of the peptides. We also do a grid-search analysis, where we fixed the Aβ42/40 ratio as baseline for *R*^2^, and then calculated the metrics of all combinations of the other peptide weights from 0 to 1 by 0.02 (either in the numerator or denominator). We did the same with PR of the controls, but the baseline ratio was Aβ37/42.

### Cross-validation

To evaluate model generalization and assess potential overfitting of wCVR, we further applied leave-one-out cross-validation (LOOCV) across all data points (mutation carriers and controls). For regression, each biomarker was fitted with a linear model to predict AAO, whereas for classification, the decision threshold in each training fold was set to the most extreme control biomarker value in the abnormal direction, prioritizing specificity for the smaller control group. Model performance was quantified using the LOOCV aggregated predictions, with the mean absolute error (MAE) for regression and the F1-score for classification. Uncertainty in point estimates was estimated via non-parametric bootstrap resampling (2000 replicates) to derive 95% CIs. Because all models were evaluated on identical holdout sets, pairwise comparisons were conducted within a paired framework: MAE differences were tested with the paired Wilcoxon signed-rank test, and classification differences were assessed using empirical two-sided *P*-values from the bootstrap distribution (2000 replicates). Multiple testing was controlled using the Benjamini–Hochberg procedure (false discovery rate 5%), with adjusted *P*-values <0.05 considered statistically significant.

### The model: weighted composite value ratio

We ran BioDisCVR^24^ to find which peptides are selected to go to the numerator, and which are selected to go to the denominator, along with their linear combination weights, obtaining a wCVR. The general formula is as follows:

$wCVR=\;\frac{\sum_{i\;\in \;X_{1}}\;k_{i}\cdot X_{1i}}{\sum_{\;j\;\in \;X_{2}}\;k_{j}\cdot X_{2j}}$ , where *X*_1_ and *X*_2_ are non-overlapping non-empty sets of the available peptides, and k is a weighting factor for each peptide. To drive the algorithm, we consider different configurations, which we indicate with the suffixes −*R* for *R*^2^, −*P* for PR AUC, and RP for both (i.e. when the objective function is the product of both metrics). The BioDisCVR framework allows for a user-chosen search algorithm. We chose to run a genetic algorithm,^25^ as described in the original paper.

### Benchmarks

Different biomarker combinations have been proposed in the field, namely Aβ42/40, Aβ37/42 and the short/long ratio, which is Aβ(37 + 38 + 40)/(42 + 43).

Additionally, we evaluated all possible combinations of ratios (180 possible combinations) where either the numerator or denominator consists of one peptide, or the addition of multiple peptides, without repetition, and conserving the different proportions of the peptides as described in Petit *et al*.

## Results

We established a novel Aβ37 ELISA that showed linearity, specificity and was not sensitive to matrix effect (Supplementary Fig. 2). We observed only minimal Aβ38 cross-reactivity, producing a signal that was 34-fold lower than the Aβ37 specific signal. We expect similar concentrations of Aβ37 and Aβ38,^8,13^ meaning the contribution of non-specific signal is negligible. Importantly, our results are comparable with our published mass spectrometry findings from the same iPSC-neuronal lines (Supplementary Fig. 2D).^13^

**Figure 1:**
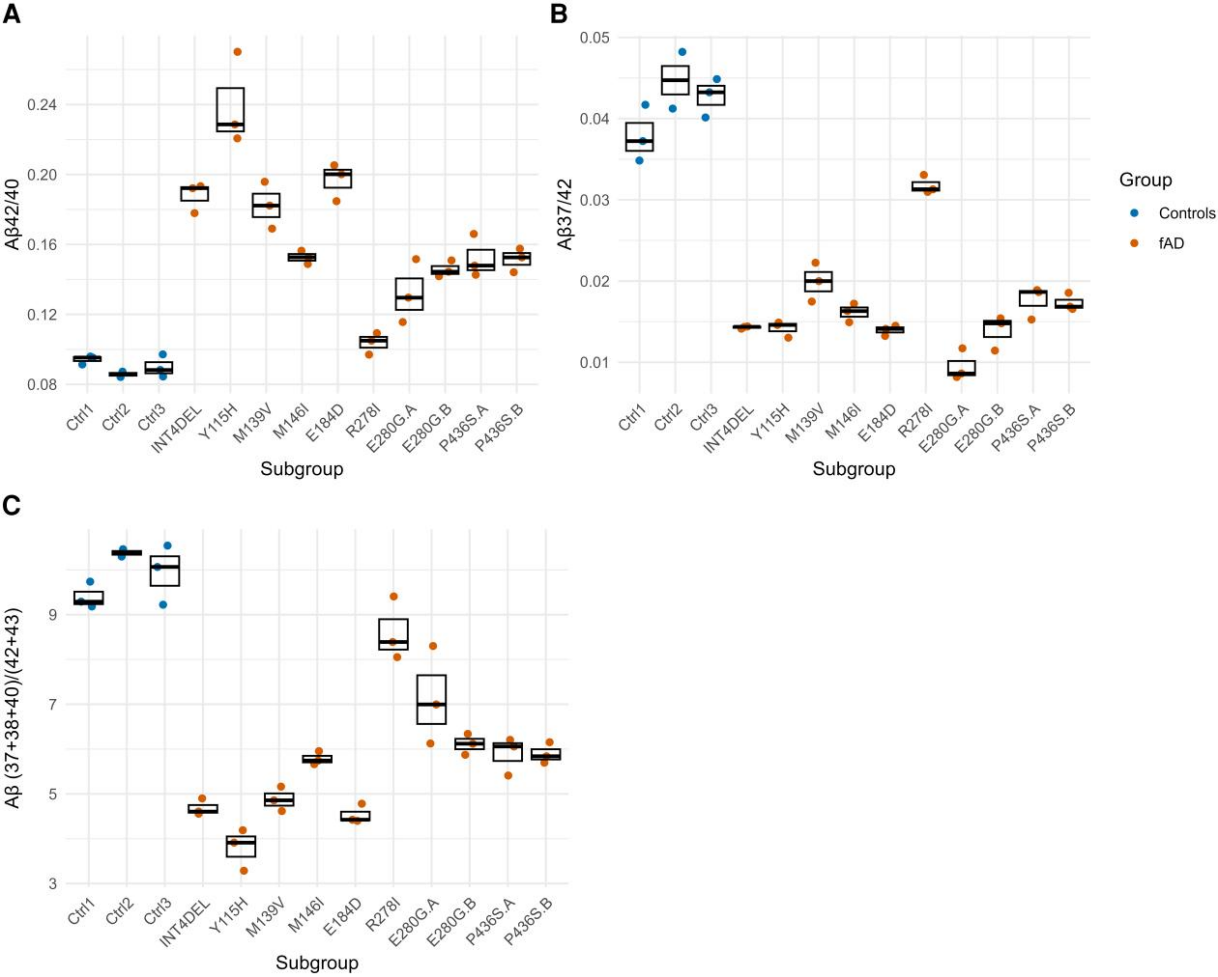
**Aβ ratios in iPSC data.** Aβ peptides were measured by ELISA showing (**A**) Aβ42:40, (**B**) Aβ37/40, and (**C**) Aβ(37 + 38 + 40)/(42 + 43) ratios. Each data point represents an independent neuronal differentiation from each iPSC line (*n* = 3 rounds of experimentation throughout, apart from Ctrl2, where *n* = 2). Control iPSC-derived neurons and *PSEN1* patient-derived iPSC-neurons are shown in blue and red, respectively. CU = cognitively unimpaired.

Figure 1 shows the proportion of Aβ37, Aβ38, Aβ40, Aβ42 and Aβ43 measured by ELISA in conditioned media from 10 *PSEN1* mutation carrying patient-derived iPSC-neuronal lines in triplicate, as well as 3 controls (data presented in Supplementary Table 3). As expected for fAD data, there is visual separation between cases and controls, as well as variation between *PSEN1* patient-derived lines.

**Figure 2:**
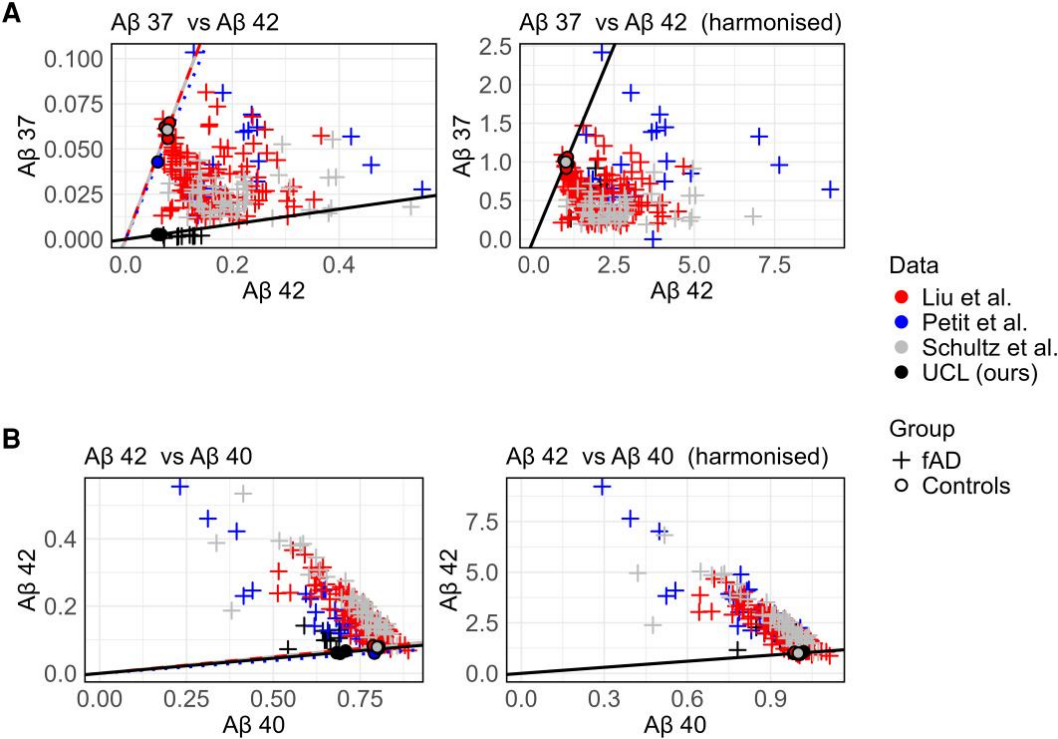
**Data harmonization and peptide distribution.** (**A**) Aβ37 versus Aβ42 and (**B**) Aβ42 versus Aβ40 ratios pre- and post-harmonization. Left: raw data, divided by total peptide count. Right: data harmonized by dividing by assay-specific average in controls. Datasets are colour coded. Lines depict the direction of data from controls, showing the successful harmonization.

To allow comparison of Aβ data from published datasets and our newly generated data (Table 1), harmonization was required because of widely acknowledged inter-assay variability (Fig. 2). Harmonization was achieved by weighting peptides based on the average relative abundance of each peptide found in control cell data within each ELISA dataset. Converting to a dimensionless, normative scale (Fig. 2 right panels) facilitates both data interpretation and combination of multi-assay datasets (Aβ peptide ratios have been shown to be highly replicable between control lines^13^). Supplementary Figure 3 shows the relative peptide amount of control cell lines for each dataset before harmonization. Although all datasets share similar general relative abundance (e.g. Aβ40 being the most abundant, and Aβ43 being the least), there are some important differences, especially for our cohort (iPSC), where the relative abundance of Aβ37 is lower and the Aβ38 is higher than the rest of the cohorts.

**Figure 3:**
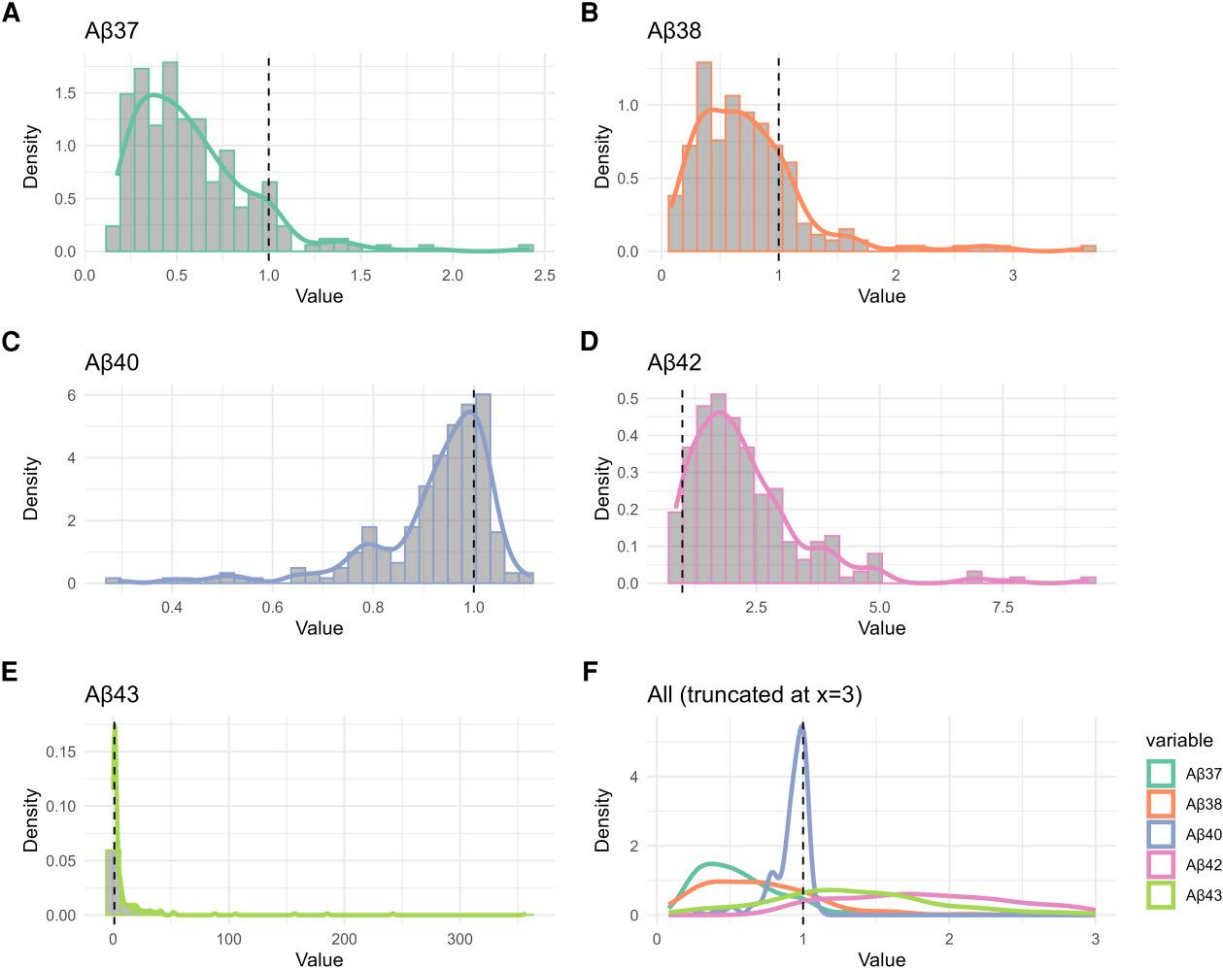
**Aβ peptide distribution from fAD data relative to controls.** Representation of all fAD data deviating from the mean of controls, set as 1. Each Aβ peptide distribution is shown; (**A**) Aβ37, (**B**) Aβ38, (**C**) Aβ40, (**D**) Aβ42 and (**E**) Aβ43, plus (**F**) all peptides combined. Most deviations of Aβ37, Aβ38 and Aβ40 occur below the controls (91.3%, 82.6% and 72.9%, respectively), while deviations of Aβ42 and Aβ43 occurred above the mean of controls (97.1% and 84.5%, respectively).

Figure 3 shows the peptide distribution of all fAD data, relative to the controls. Most deviations of Aβ37, Aβ38 and Aβ40 occur below the mean of controls (91.3%, 82.6% and 72.9%, respectively), while most deviations of Aβ42 and Aβ43 occurred above the mean of controls (97.1% and 84.5%, respectively).

Table 2 shows the correlation (Pearson and Kendall) with the AAO for all peptides, per dataset (pre-transformation), and for all data merged (post-transformation). The measures are all relative to the sum of the five peptides. All the statistically significant comparisons (*P* < 0.05, in bold, and thus the subset of corrected *P*-values) share the same message: positive correlation for Aβ37, Aβ38 and Aβ40, and negative correlation for Aβ42 and Aβ43.

**Table 2:** Correlation measures, per peptide, per cohort (and all data ‘all’), with age-at-onset.

		Pearson				Kendall	
	Peptide	Ρ	*P*-value	Lower_CI	Upper_CI	Τ	*P*-value
**iPSC**	Aβ37	0.114	0.754	−0.555	0.694	−0.068	0.787
	Aβ38	−0.156	0.666	−0.716	0.525	−0.068	0.787
	Aβ40	0.462	0.179	−0.237	0.846	0.296	0.241
	Aβ42	−0.559	0.093	−0.879	0.109	−0.341	0.176
	Aβ43	0.358	0.310	−0.351	0.806	0.159	0.528
**Petit**	Aβ37	−0.141	0.512	−0.515	0.279	−0.167	0.268
	Aβ38	−0.093	0.666	−0.478	0.323	−0.022	0.902
	Aβ40	**0**.**781**	**0**.**000**	0.551	0.900	0.534	**0**.**000**
	Aβ42	**−0**.**834**	**0**.**000**	−0.926	−0.650	−0.710	**0**.**000**
	Aβ43	−0.292	0.167	−0.622	0.127	−0.241	0.101
**Liu**	Aβ37	**0**.**253**	**0**.**004**	0.085	0.407	0.172	**0**.**004**
	Aβ38	**0**.**194**	**0**.**026**	0.024	0.354	0.190	**0**.**001**
	Aβ40	**0**.**486**	**0**.**000**	0.343	0.607	0.342	**0**.**000**
	Aβ42	**−0**.**641**	**0**.**000**	−0.732	−0.528	−0.521	**0**.**000**
	Aβ43	**−0**.**238**	**0**.**006**	−0.394	−0.070	−0.144	**0**.**016**
**Schultz**	Aβ37	0.043	0.751	−0.222	0.303	0.092	0.319
	Aβ38	−0.007	0.958	−0.270	0.256	0.187	**0**.**043**
	Aβ40	**0**.**467**	**0**.**000**	0.233	0.650	0.334	**0**.**000**
	Aβ42	**−0**.**581**	**0**.**000**	−0.732	−0.376	−0.430	**0**.**000**
	Aβ43	0.001	0.995	−0.262	0.264	0.063	0.497
**All**	Aβ37	0.031	0.644	−0.101	0.163	0.074	0.104
	Aβ38	0.066	0.330	−0.067	0.196	0.103	**0**.**024**
	Aβ40	**0**.**493**	**0**.**000**	0.386	0.587	0.343	**0**.**000**
	Aβ42	**−0**.**580**	**0**.**000**	−0.661	−0.485	−0.447	**0**.**000**
	Aβ43	−0.108	0.109	−0.237	0.024	−0.095	**0**.**038**

In bold, uncorrected *P*-values below 5%

Using the full harmonized dataset, we examined whether the position of the *PSEN1* mutation is associated with either age at onset or the Aβ42/40 ratio (Supplementary Fig.  4A and 4B, respectively). For age at onset, the Kendall’s *τ* was 0.0091 (*z* = 0.200, *P* = 0.841) and the Pearson correlation was 0.0051 (*t* = 0.076, *df* = 221, *P* = 0.940), indicating no evidence of a monotonic or linear association. For the Aβ42/40 ratio, the Kendall’s *τ* was −0.0504 (*z* = −1.115, *P* = 0.265) and the Pearson correlation was −0.0533 (*t* = −0.793, *df* = 221, *P* = 0.428). The practically zero coefficients and high *P*-values suggest that the observed correlation is not statistically significant. Together, these analyses show no detectable relationship between mutation position and either clinical or biochemical measures.

**Figure 4:**
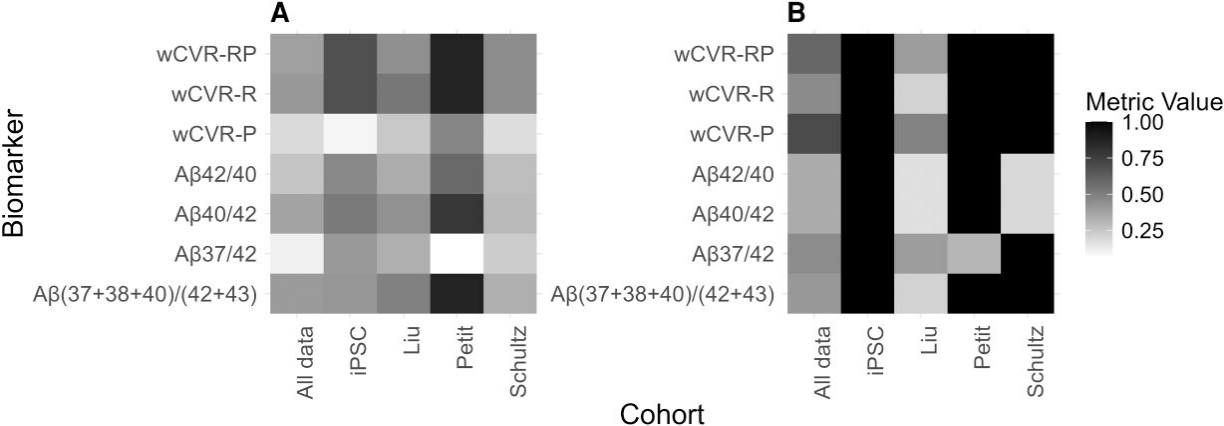
**Biomarker performance heatmap.** Performance is shown for (**A**) age-at-onset correlation (R2) and (**B**) case–control classification (Precision-Recall AUC). Rows represent biomarkers and columns represent cohorts (iPSC represents data generated in this study); darker grayscale indicates superior performance (scale 0–1). The weighted composite value ratios (wCVR) consistently perform well across both metrics. Differences in intensity across columns reflect the biological heterogeneity of fAD mutations within each cohort. Abbreviations: AAO, age-at-onset; wCVR, weighted composite value ratio (suffixes denote optimization for: P, classification (wCVR trained for case–control classification); R, AAO correlation (wCVR trained for predicting mutation-associated onset); RP, both).

A dataset-specific evaluation of biomarkers is shown as heatmaps in Figure 4. We compare established ratios (Aβ42/40, Aβ40/42, Aβ37/42 and Aβshort/long) with wCVR analyses with different configurations (see Methods), where wCVR was optimized per cohort. For both correlation with AAO (coefficient of determination, Fig. 4, left panel) and case–control classification (PR, Fig. 4, right panel), the heatmaps show performance heterogeneity between the cohorts. Data from iPSC, Petit or Schultz classify well (perfect classification for all biomarkers except for Aβ42/40, Aβ37/42 and Aβ40/42), and data from Petit exhibits the highest coefficient of determination, followed by iPSC. As for the novel biomarkers, wCVR achieves superior performance within each metric to which it is directed, namely wCVR-P achieves the best classification for PR of the controls, and wCVR-R shows the best correlation with AAO when driven by the coefficient of determination. Aβshort/long and Aβ40/42 also perform well for correlation with AAO.

**Figure 5:**
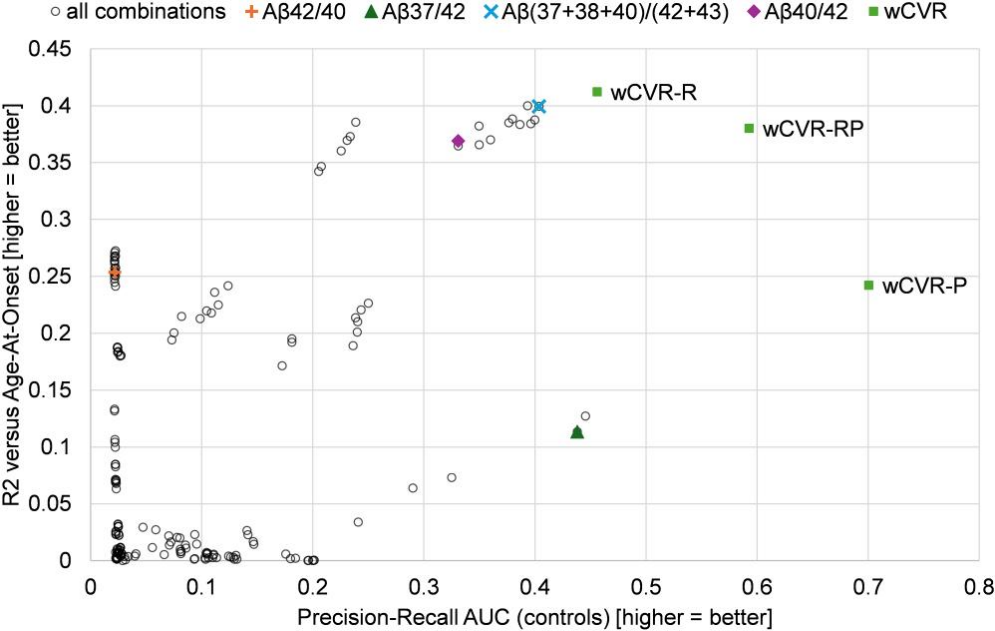
**Performance landscape of Aβ ratio biomarkers.** The scatter plot compares Age-at-Onset correlation (R2, *y*-axis) against case-control classification performance (Precision-Recall AUC, *x*-axis), using all available harmonized data (measures of Aβ peptides 37, 38, 40, 42 and 43). Empty circles represent all possible distinct combinations of non-weighted ratios; coloured markers highlight commonly used reference ratios. The weighted composite biomarkers (wCVR, green squares) consistently occupy the upper-right quadrant, indicating superior combined performance. Notably, the distribution of wCVR points reveals a Pareto frontier, illustrating a trade-off between metrics: wCVR-P is trained to maximize Precision-Recall-AUC for case–control separation and is shifted right (higher AUC), while wCVR-R is trained to maximize R2 with age at onset and is shifted up (higher R2), and wCVR-RP is jointly optimized for R2 and PR-AUC.

Figure 5 shows the results of our ratio biomarker experiments. The map of *R*^2^ (AAO) versus PR AUC shows wCVR biomarkers towards the upper right quadrant, demonstrating superior performance to most simple combinations of peptides. The literature biomarkers also do well in one metric each, but not both: Aβshort/long shows high *R*^2^, and Aβ37/42 outperforms most simple ratios in PR AUC.

Table 3 contains the quantification of the performance of the salient biomarkers, with 95% CIs from bootstrapping (see Methods). It is noteworthy that wCVR is able to improve on established ratios from the literature; however, different wCVR biomarkers excel at PR versus coefficient of determination.

**Table 3:** Evaluation metrics for the biomarkers considered

Biomarker	AUC	PR AUC controls	PR AUC fAD	R^2^
wCVR-R	0.977 (0.953, 0.994)	0.456 (0.287, 0.805)	0.999 (0.998, 1.000)	**0.412** (**0.284, 0.526)**
wCVR-RP	0.986 (0.966, 0.997)	0.593 (0.363, 0.934)	0.999 (0.999, 1.000)	0.380 (0.255, 0.497)
wCVR-P	**0.992** (**0.977, 1.000)**	**0.700** (**0.435, 1.000)**	**1.000** (**0.999, 1.000)**	0.242 (0.145, 0.350)
Aβ42/40	0.961 (0.929, 0.983)	0.345 (0.217, 0.615)	0.998 (0.997, 0.999)	0.259 (0.187, 0.343)
Aβ37/42	0.977 (0.953, 0.993)	0.454 (0.29, 0.785)	0.999 (0.998, 1.000)	0.114 (0.048, 0.230)
short/long	0.974 (0.947, 0.991)	0.411 (0.265, 0.729)	0.999 (0.998, 1.000)	0.403 (0.275, 0.517)

In bold, the best result per metric. wCVR = weighted composite value ratio, which is a linear combination of peptides both in the numerator and denominator. The suffix -R indicates that the wCVR was optimized for *R*^2^ with respect to age-at-onset; -P indicates that wCVR was optimized to maximize the PR AUC for the controls; -PR indicates that wCVR was optimized to maximize the product of both metrics (regression with age-at-onset and classification for the controls). AUC = area under the receiver operator characteristic curve. PR AUC = area under the precision-recall curve. fAD = data from familial Alzheimer’s disease (mutation-carriers). R^2^ = coefficient of determination, using Pearson’s correlation with age-at-onset.

Table 4 lists the peptide weights (denominator as negative numbers, bold and underlined) for the different wCVR configurations, along with their classification and regression performance. There is a trade-off between the two evaluation metrics (classification and regression), which is determined by the weight shifting and position in the ratio of the peptides. In all cases, Aβ40 is in the numerator and Aβ42 and Aβ43 are in the denominator. Aβ38 is actively (10–19%) in the numerator when the objective function includes the coefficient of determination, and Aβ37 appears in the numerator when classification (PR) is considered. When considering both metrics of classification and regression, the composite value ratio becomes similar to the short/long ratio, with notable more weighting of the Aβ42 in the denominator, and Aβ40 in the numerator. Table 4 also shows the wCVR for the individual datasets, demonstrating the different contributions of each peptide to the combined performance (PR plus coefficient of determination) for each dataset. Note that Aβ42 and Aβ43 are consistently in the denominator, while Aβ37, Aβ38 and Aβ40 are in the numerator.

**Table 4:** wCVR weights per configuration (all data)

Data-driven weighting and combination of peptides (wCVR)							
Aβ37	Aβ38	Aβ40	Aβ42	Aβ43	biomarker	PR AUC	R^2^
***−0***.***2%***	19.0%	81.0%	***−97***.***8%***	***−2***.***0%***	wCVR-R	0.456	0.412
21.0%	9.8%	69.2%	***−93***.***6%***	***−6***.***4%***	wCVR-RP	0.593	0.382
88.9%	***−27***.***8%***	11.1%	***−59***.***0%***	***−13***.***2%***	wCVR-P	0.705	0.185

Short/long relative weights per cohort (pre-harmonisation)					
Aβ37	Aβ38	Aβ40	Aβ42	Aβ43	Cohort
0.3%	25.7%	74.1%	***−99***.***7%***	***−0***.***3%***	**iPSC**
3.7%	13.0%	83.3%	***−99***.***4%***	***−0***.***6%***	**Petit**
6.6%	6.7%	86.7%	***−98***.***6%***	***−1***.***4%***	**Liu**
6.6%	6.6%	86.8%	***−98***.***6%***	***−1***.***4%***	**Schultz**

Suffix -R optimizes wCVR for *R*^2^ versus age-at-onset. Suffix -P optimizes wCVR versus precision-recall area under the curve (PR AUC). Suffix -RP optimizes for the multiplication of both *R*^2^ and PRAUC. wCVR = weighted composite value ratio, which is a linear combination of peptides both in the numerator and denominator. Values for the denominator are negative, and distinguished as bold, italicized and underlined.

Results from a permutation feature importance analysis are shown in Supplementary Table 2. For all metrics, and for both displayed biomarkers (Aβshort/long and wCVR-RP), Aβ42 is the peptide providing the biggest impact. The data-driven wCVR-RP better employs information from Aβ43, as the importance values are higher in all metrics.

Different patterns were observed, depending on the metric used for optimization, when bootstrapping the aggregated data and running BioDisCVR three times per bootstrap to get the best wCVR. Supplementary Figure 5 shows the boxplots of the weights for 200 bootstrapped samples, where a positive weight places the peptide in the numerator, and a negative weight in the denominator of the ratio. However, these weights were obtained by fitting different resampled data, so to better understand their effect, Supplementary Figure 6 shows the evaluation metrics against peptide weights. In all cases, we can observe a clear convex space of data points, with a slight truncation for the precision-recall plots. A similar pattern of an optimal weight combination can be observed in Supplementary Figure 7, where the grid-search analysis of weighted combinations shows a single peak of performance (yellow). These data suggest that employing multiple peptides improves performance in each case.

Across all biomarkers, LOOCV demonstrated high classification performance and similar performance in predicting age at onset. Among the tested measures, wCVR-RP achieved the highest overall performance, with an F1-score of 0.984 (95% CI: 0.972–0.995) and the lowest MAE of 6.205 years (95% CI: 5.601–6.823). Aβ37/42 also performed strongly (F1-score 0.982, MAE 6.892), followed by Aβ42/40 (F1-score 0.963, MAE 6.850). In contrast, the short/long ratio showed substantially lower classification performance (F1-score 0.857) and higher prediction error (MAE 7.706). Pairwise comparisons confirmed that wCVR-RP significantly outperformed short/long for both classification (adjusted *P* < 0.0001) and regression (adjusted *P* < 0.0001). Compared with Aβ42/40 and Aβ37/42, wCVR-RP yielded significantly lower MAE (adjusted *P* = 0.016 and 0.0007, respectively), although differences in F1-score were not statistically significant after correction (adjusted *P* = 0.15 and 0.91, respectively). Together, this demonstrates the robustness of wCVR in both classification (mutation detection) and regression (AAO prediction).

## Discussion

We measured five neuronal Aβ peptides from 10 patient-derived iPSC lines to investigate Aβ ratios in an unbiased, data-driven manner. The data independently verify a differential contribution of longer peptides (Aβ42 and Aβ43) and shorter peptides (Aβ40, Aβ38 and Aβ37) to disease. We explored the relative contribution of each peptide to disease classification and onset prediction. Aβ40 and Aβ42 represent the major contributing factors; however, the inclusion of additional peptides further improves the model. Notably, the weightings were distinct when optimizing for classification versus onset prediction, suggesting differential relevance of Aβ peptides. Finally, we observed that data from iPSC models show greater weighting for Aβ38 and less for Aβ40 compared with data from *PSEN1/2* double-knockout cell lines.^8-10^

Employing a patient-derived iPSC-neuronal model, this study complements previous studies in *PSEN1*/*PSEN2* double-knockout cell lines due to the presence of one healthy *PSEN1* allele and two healthy *PSEN2* alleles, analogous to the patient setting. This model, therefore, represents a physiological model of Aβ production in human neurons, which we have previously shown to faithfully correlate with clinical data from the same donor.^6,13^

We developed a novel Aβ37 indirect ELISA that showed sensitivity and specificity. Some minor cross-reactivity to Aβ38 was observed, which should be considered with future adoption. Similar (non-identical) relative abundance of Aβ37 was observed when measured by ELISA and mass spec, giving us further confidence in this assay, as slight differences in detection between techniques are commonly observed when quantifying Aβ^13^. Widespread adoption of this assay is required to independently validate the assay.

Previously reported ratios performed well: Aβ37:42^8^ distinguished patient and control lines better than other linear ratios, and Aβshort/long^9^ performed best for predicting AAO. However, wCVR were able to outperform linear ratios in both metrics, suggesting that increasing the number of Aβ peptides and assigning weight to each is able to further improve biomarker performance. The optimal ratio for all data, centred around both disease classification and predicting AAO is: wCVR=(21⋅Aβ37+10⋅Aβ38+69⋅Aβ40)/(94⋅Aβ42+6⋅Aβ43). Data-driven analyses support the increased pathogenicity of Aβ42 and Aβ43 versus Aβ37, Aβ38 and Aβ40; supporting *in vitro* data such as the differential aggregation kinetics of the longer species compared to the shorter species.^26^

Robustness was confirmed by LOOCV, which showed that wCVR-RP consistently achieved the lowest prediction error and highest classification performance. It significantly outperformed the short/long ratio across both tasks and yielded lower MAE than Aβ42/40 and Aβ37/42. While classification differences with the linear ratios were not significant after correction, the consistent gain in regression performance underscores the reliability of wCVR as a predictor of age at onset.

This approach is clearly translational, and represents a pipeline that can be reiterated for larger experimental or clinical datasets to develop weighted peptide ratios that greatly improve diagnostic and predictive Aβ biomarkers in CSF and plasma. We believe that the approach will be generalizable to sporadic and late-onset forms of AD (LOAD), through the measurement of multiple Aβ species in blood plasma. A first step on this path would be to test the five Aβ peptides in iPSC models of LOAD, such as those developed in the literature,^27,28^ and patient-derived blood plasma. As yet, these data do not exist. Aβ abundance in plasma represents a readout on APP processing and Aβ *production*, whereas Aβ in CSF informs on the degree of Aβ *aggregation* in the brain and the sequestration of longer species. Although Aβ ratios have been shown to be altered in plasma, the degree of change is much smaller than the changes observed in CSF.^29^ Therefore, the use of weighted ratios will greatly improve diagnostic capabilities in plasma. wCVR may also be applicable to improve upon biomarker performance in CSF as a measure of Aβ aggregation in fAD and LOAD. It will be interesting to employ the methods presented herein to uncover specific wCVR weightings for Aβ species in CSF from LOAD and fAD. Further, this approach can be broadened to any number of biomarker measures to distinguish groups from a control population.

A diversity of Aβ peptides exists, and fAD mutations alter their relative abundance differently. We analysed wCVR to infer the relative contribution of each peptide to classifying disease and for predicting age at onset. The fact that weights were dissimilar for the two biomarker analyses suggests that ‘pathogenicity’ and ‘severity’ show some distinction, for example changes to Aβ38 can inform on onset prediction but is negatively associated with classification. Additionally, we observed differences in the wCVR in iPSC-models compared to cell models, such that Aβ38 has a higher weighting at the expense of Aβ40. This may inform on the presence of a healthy *PSEN1* allele in iPSC models, speaking to the overrepresentation of Aβ40 compared with other peptides.

*PSEN1* mutation carriers show a diversity in the onset of fAD, even within families with the same mutation. Our analyses find no association between the location of the mutation across the PSEN1 protein and AAO, similar to previous observations.^10^ These data suggest that the severity of each mutation depends upon the biochemical effect of each amino acid substitution on the tertiary/quaternary protein structure.

Our study comes with some limitations to consider. One limitation is the low sample sizes, although we have strived for robust replication, via triplicate neuronal differentiations and 10 patient-derived lines. More donors and data replicates will further improve the confidence in these findings, for which independent studies are required. One consequence of the low sample size is the limitation in complexity for the harmonization pre-process; ideally, we would consider the spread of control measures on top of their magnitude. As it currently stands, the harmonization may amplify noise in low-abundance peptides such as Aβ43. However, since the wCVR learns weighted average combinations of peptides, this problem ceases to be in our algorithm. It would only be problematic if we were to do simple addition, which does not happen in this work. A further limitation to consider is that different ELISA methods to the same peptide may introduce variation (e.g. differential detection of monomers, oligomers or non-specific binding). However, given the literature stating that relative Aβ abundance is remarkably consistent in control cells, the harmonization approach adopted here controls for such variation. This assertion would support multi-site adoption of wCVR, but requires empirical validation.

Another limitation is a lack of female control lines; however, we predict *PSEN1* mutations to have a dominant effect on γ-secretase processivity irrespective of gender. Additionally, the panel of iPSC samples tested comprise an over-representation of mutations that produce relatively high levels of Aβ43 (R278I,^13^ E280G^14^ and P436S^15^), potentially skewing the relative contribution of Aβ43. However, our analysis of other datasets, which do not have the same issue, mitigates this limitation. Finally, AAO is known to be variable, and so the regression analyses should be considered with this caveat in mind.

## Conclusions

In conclusion, this study presents new data, a new fluid biomarker harmonization method, and a new ratio-based biomarker that incorporates feature importance in a transparent manner. Our experimental results demonstrate superior performance to the previous state-of-the-art ratio biomarkers, which have previously demonstrated that Aβ ratios correlate with cognitive measures,^9,10^ for example, Aβ processing was shown to correlate with cognitive symptom manifestation and decline (mini-mental state examination, clinical dementia rating—sum of boxes, and Wechsler Memory Scale—Revised Logical Memory Delayed Recall scores^10^). Together, we have shown that our new approaches to harmonization and for incorporating extra peptides in the ratio can be a robust complement to emerging plasma biomarkers, with the potential to refine risk stratification, support the diagnosis of novel fAD mutations, and accelerate the translation of Aβ-based assays into clinical practice.^29^

## Supplementary Material

fcag105_Supplementary_Data

## Data Availability

The datasets generated and analysed during the current study are available in the supplementary figures and from the primary publications: Petit *et al.* colleagues,^9^ Liu *et al.*,^8^ and Schultz *et al*.^10^ The framework code used for biomarker discovery (weighted-composite value ratios), is available in a public GitHub repository under the GNU General Public License v3.0: https://github.com/isaac-6/biodiscvr.
