# Supplementary material for: Unbiased data-driven analysis of five amyloid-beta peptides for biomarker investigations in familial Alzheimer’s disease
Source: Brain Commun. 2026 Mar 22;8(2):fcag105. doi: 10.1093/braincomms/fcag105 (PMC13096935; doi:10.1093/braincomms/fcag105)
Supplement: fcag105_Supplementary_Data [file fcag105_supplementary_data.docx]

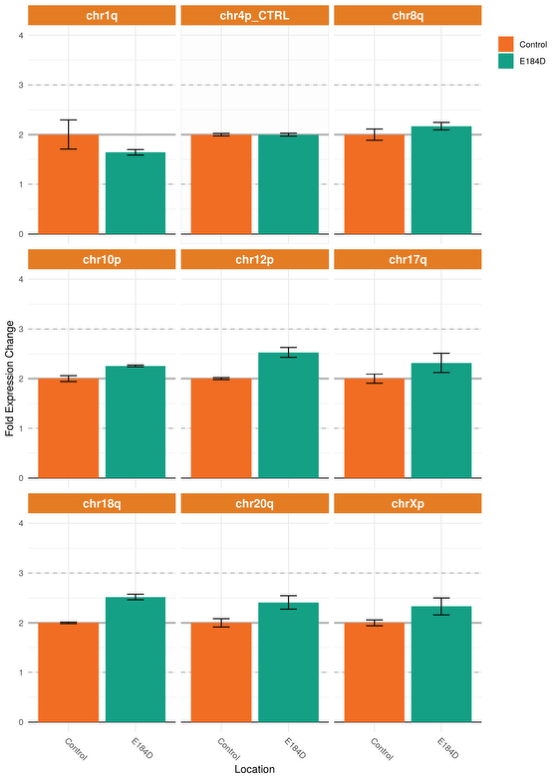


C

D


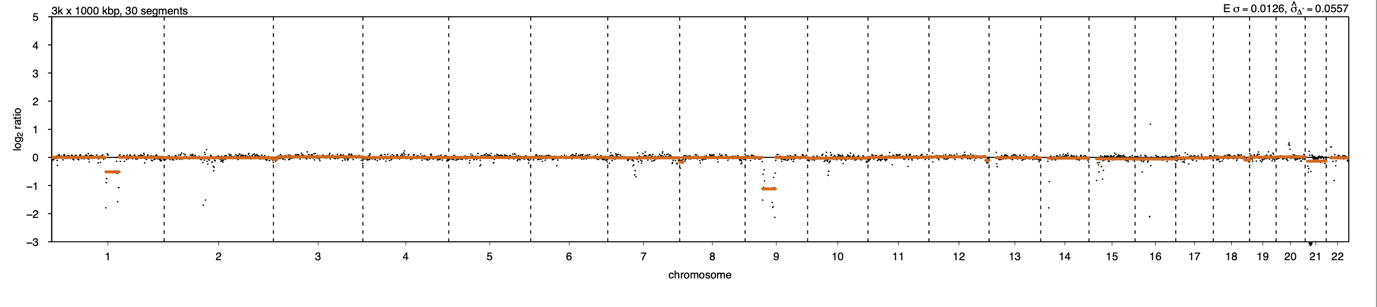


**Supplementary Figure 1. Characterisation of newly generated *PSEN1* E184D iPSC line.** A) Immunocytochemistry of iPSCs for pluripotency markers NANOG and SSEA4. B) Sanger sequencing to confirm the point mutation Chr14:73659355 A>C. C) qPCR data of commonly duplicated or deleted genomic regions to support stable karyotype. D) Low coverage whole genome sequencing to confirm karyotype stability.

**Supplementary Figure 2. Characterisation of novel Aβ37 ELISA.** A) Linearity is shown using a serial dilution of recombinant Aβ37. The matrix (iPSC-neuron conditioned cell culture media) does not affect linearity of recombinant Aβ standards, evidenced by all datapoints raised by the same degree (corresponding to Aβ37 in the conditioned media). B) Representation of matrix effect plotted as a % recovery. C) Measures of specificity against other recombinant Aβ peptides. The Aβ37 antibody shows some cross-reactivity with Aβ38, however this is 34-fold lower (right shift). D) Comparison of Aβ37 quantification relative to Aβ42 from newly generated ELISA data with published mass spec data (Supplementary Table 4)^1^.


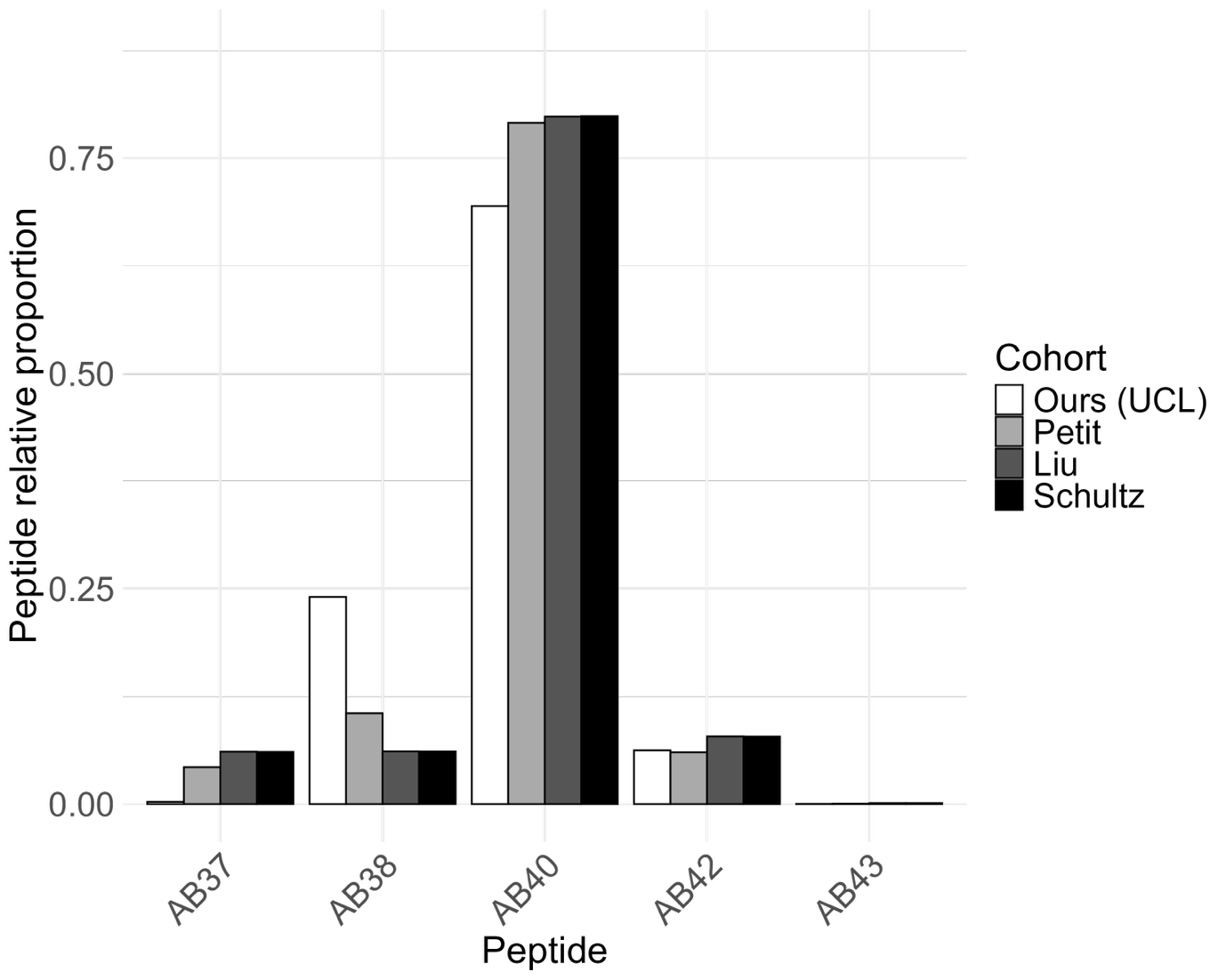


**Supplementary Figure 3. Peptide abundance profiles.** Peptide abundance (fraction) per cohort (in grayscale) is shown on the vertical axis for Aβ37, Aβ38, Aβ40, Aβ42, and Aβ43. UCL data had an over-representation of Aβ38 compared to the other cohorts.

**
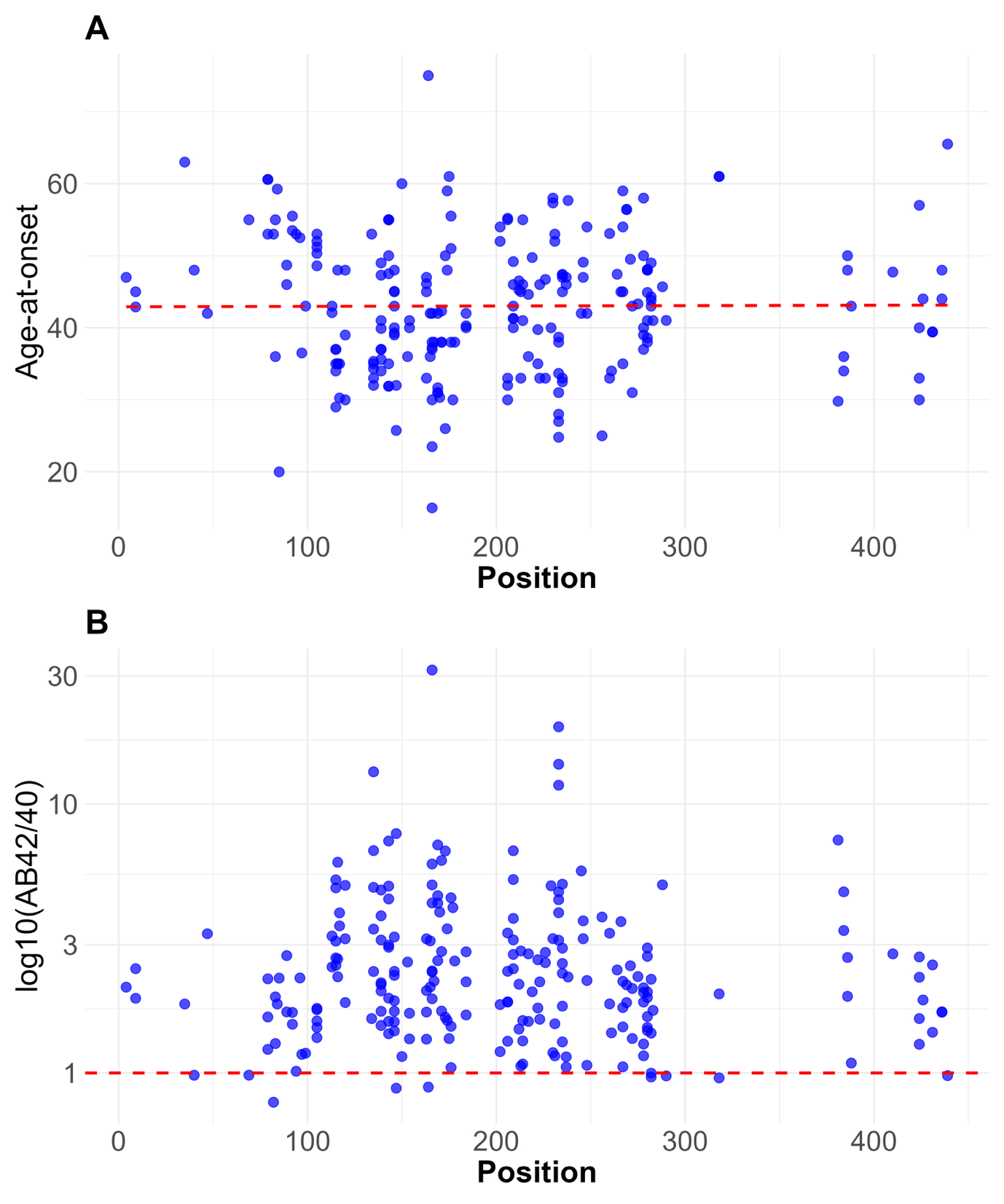
**

**Supplementary Figure 4. Lack of association between PSEN1 mutation position with age at onset and Aβ42:40 ratio across harmonised data.**

(A) Age at onset (y-axis) plotted against mutation position in PSEN1 peptide (x-axis). The red dashed line represents the linear best‑fit regression line. Kendall's τ = 0.0091 (z = 0.200, p = 0.841); Pearson r = 0.0051 (t = 0.076, df = 221, p = 0.940). (B) log₁₀-transformed Aβ42:40 ratio (y-axis) plotted against mutation position in PSEN1 (x-axis). The red dashed line marks the ratio of controls as reference. Kendall's τ = -0.0504 (z = -1.115, p = 0.265); Pearson r = -0.0533 (t = -0.793, df = 221, p = 0.428). The coefficients being close to zero with high p-values suggest that the observed correlations are not significantly significant.


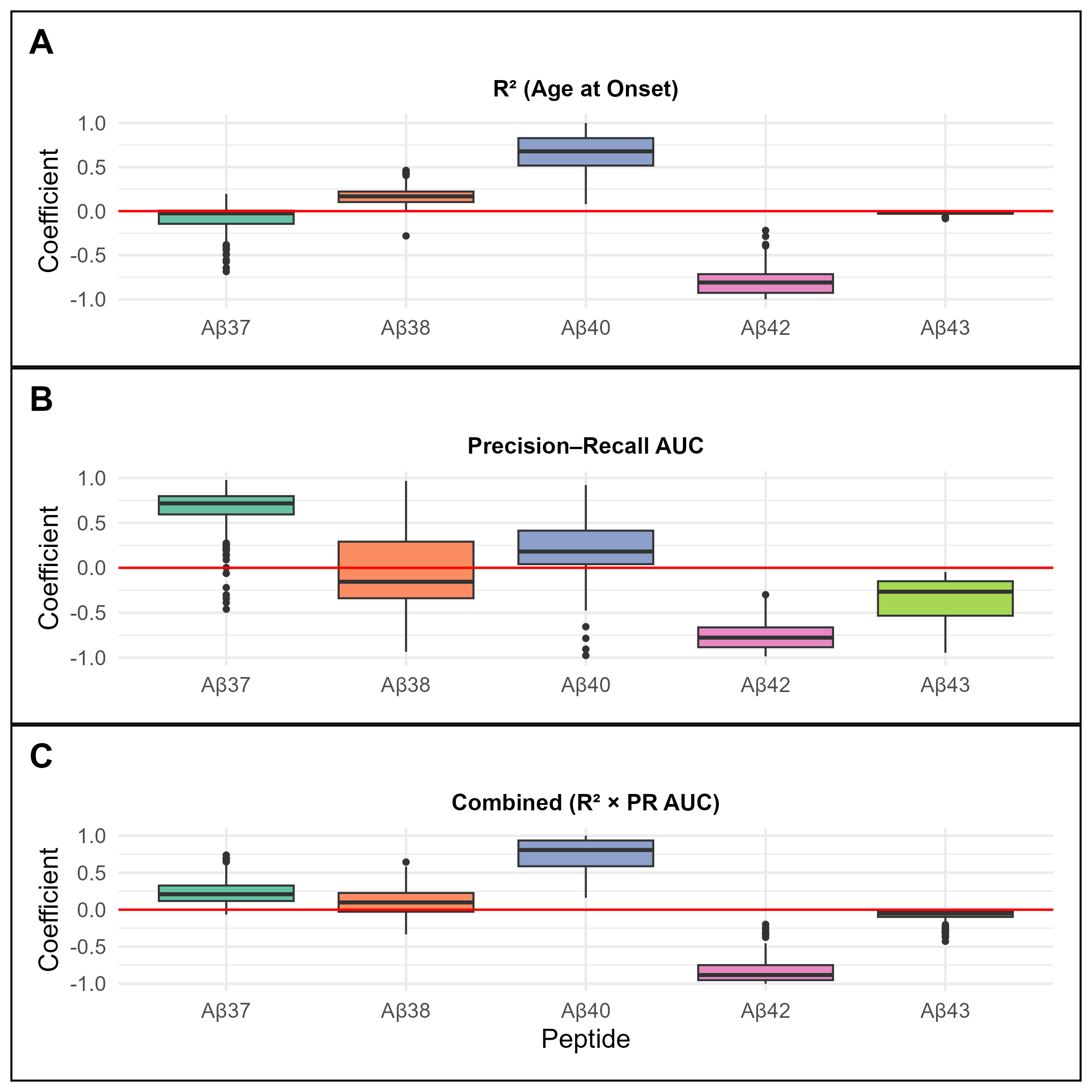


**Supplementary Figure 5. Peptide importance in weighted composite value ratios (wCVR) across 200 bootstrap iterations.**

Boxplots show the distribution of peptide weights (0–1) assigned during bootstrap optimisation, signed to indicate numerator (+) or denominator (–) placement in the wCVR ratio. Three configurations are shown:

A) Optimisation based on R² with age at onset (Fitness = R²). The peptides Aβ40 (numerator) and Aβ42 (denominator) dominate.

B) Optimisation based on precision–recall AUC for case–control classification. The peptides Aβ37 (numerator) and Aβ42 (denominator) dominate.
C) Joint optimisation of both metrics (Fitness = R² × PR_AUC) produces a hybrid wCVR dominated by Aβ40/Aβ42, but with Aβ37 contributing positively (numerator).
The red horizontal line at y = 0 separates numerator and denominator contributions.

**
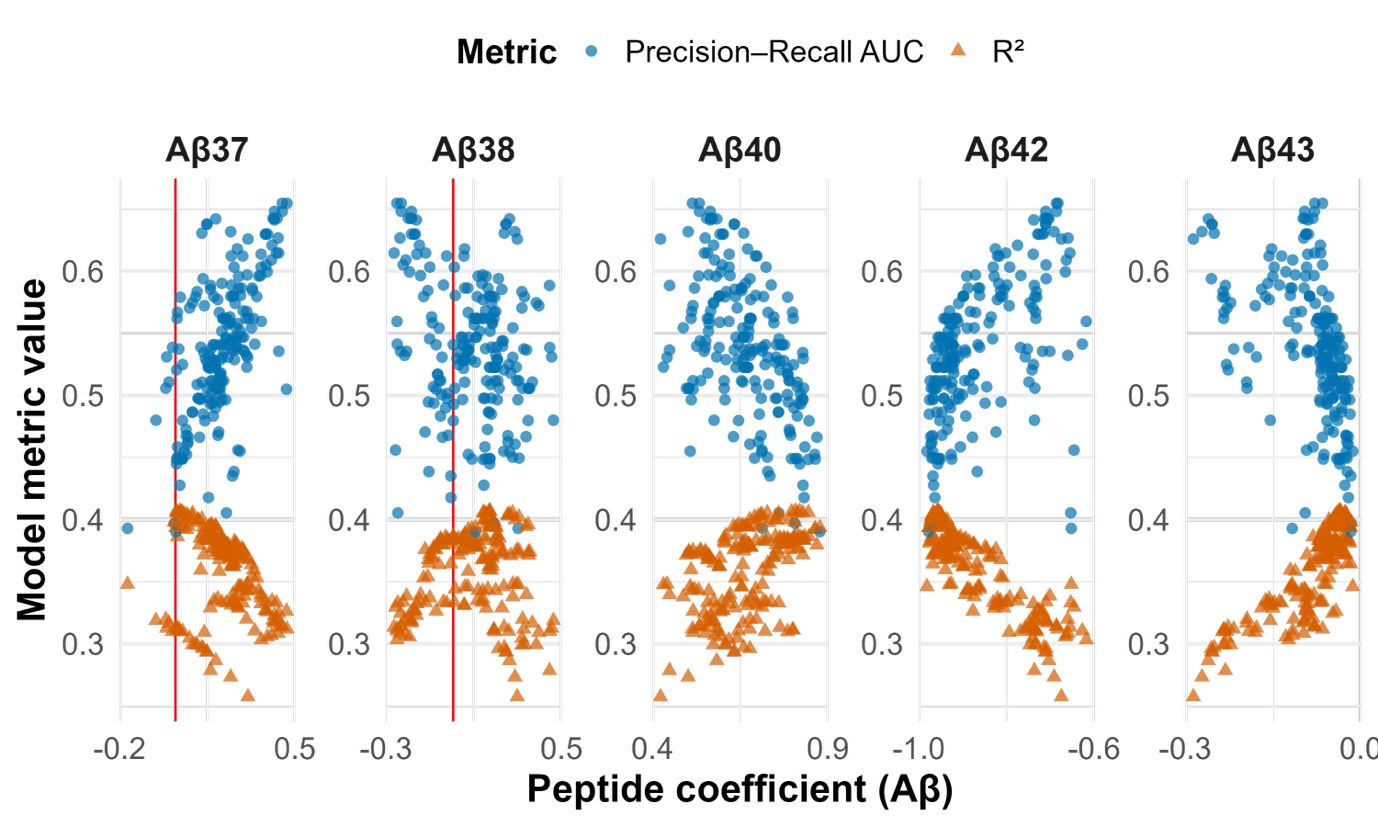
**

**Supplementary Figure 6. Biomarker performance by peptide weight across 200 bootstrap iterations.**

Each panel shows biomarker performance (vertical axis) as a function of the estimated weight assigned to a given Aβ peptide (horizontal axis). Each point corresponds to one of 200 bootstrap experiments in which the ratio of a weighted linear combination of peptides was optimised to jointly maximise two multiplicative metrics: coefficient of determination (R²) with age‑at‑onset and precision–recall area under the curve (PR‑AUC). Blue circles denote R² values; orange triangles denote PR‑AUC values. The red vertical line marks weight = 0; positive weights place the peptide in the numerator of the ratio, and negative weights place it in the denominator. Across peptides, the convex arrangement of points indicates a consistent optimal weighting region, supporting the contribution of multiple peptide combinations to improved biomarker performance.

**Supplementary Figure 7. Biomarker performance by peptide weight (grid search).** Left: wCVR R^2^ with AAO. Right: precision-recall AUC. The colour gradient shows a sweet spot where biomarker performance is improved by adding other peptides to either Aβ40/Aβ42 (left) or Aβ37/Aβ42 (right).

**Supplementary Table 1. iPSC lines used in this study**

| Line | Details and reference | Female/Male | Age at biopsy | APOE status | Age at onset |
| --- | --- | --- | --- | --- | --- |
| Control 1 | KOLF2.1J ^2^ ​ | Male | 55-59 | 3/3 | - |
| Control 2 | ND41886 ^1^ | Male | 64 | 2/3 | - |
| Control 3 | RBi01 (Sigma) ^1^ | Male | 45-49 | 3/3 | - |
| *PSEN1* int4del | EBiSC ^1^ | Female | 47 | 3/3 | 47 |
| *PSEN1* Y115H | *PSEN1* Y115H ^1^ | Male | 39 | 3/3 | 34 |
| *PSEN1* M139V | EBiSC ^1^ | Female | 45 | 2/3 | 34 |
| *PSEN1* M146I | EBiSC ^1^ | Male | 38 | 3/3 | - |
| *PSEN1* E184D | This study | Female | 43 | 3/3 | 45 |
| *PSEN1* R278I | *PSEN1* R278I cA ^1^ | Male | 60 | 2/4 | 58 |
| *PSEN1* E280G #1 | *PSEN1* E280G.A ^3^ | Male | 45 | 3/3 | 41 |
| *PSEN1* E280G #2 | *PSEN1* E280G.B ^3^ | Male | 49 | 3/4 | 38 |
| *PSEN1* P436S #1 | *PSEN1* P436S #1 ^4^ | Female | 52 | 2/3 | 48 |
| *PSEN1* P436S #2 | *PSEN1* P436S #2 ^4^ | Female | 48 | 3/3 | 44 |

**Supplementary Table 2: Permutation feature importance analysis.** In bold, the peptide with the biggest effect. Both biomarkers (short/long and wCVR-RP) have a similar composition: the peptides Aβ37, 38 and 40 in the numerator, and Aβ42 and 43 in the denominator.

|  | **Feature importance (90% confidence interval)** | | | |
| --- | --- | --- | --- | --- |
|  | **1 - Precision-recall AUC** | | **1 - (R2 vs AAO)** | |
|  | **short/long** | **wCVR-RP** | **short/long** | **wCVR-RP** |
| Aβ37 | 0.99 (0.98, 1.04) | 1.13 (1.02, 1.19) | 1.00 (0.99, 1.00) | 0.99 (0.97, 1.02) |
| Aβ38 | 1.10 (1.01, 1.17) | 1.09 (1.06, 1.19) | 1.05 (1.02, 1.09) | 1.03 (1.00, 1.05) |
| Aβ40 | 1.06 (0.92, 1.14) | 1.18 (1.00, 1.24) | 1.06 (1.05, 1.09) | 1.06 (1.05, 1.08) |
| **Aβ42** | **1.58 (1.56, 1.61)** | **1.99 (1.82, 2.09)** | **1.61 (1.54, 1.66)** | **1.52 (1.45, 1.57)** |
| Aβ43 | 1.02 (1.01, 1.19) | 1.55 (1.18, 1.57) | 1.03 (1.00, 1.05) | 1.19 (1.10, 1.22) |

**Supplementary Table 3. Aβ measurement data**

|  |  |  | BATCH ID | AAO | Ab37 pg/ml | Ab38 pg/ml | Ab40 pg/ml | Ab42 pg/ml | Ab43 pg/ml |
| --- | --- | --- | --- | --- | --- | --- | --- | --- | --- |
| Experiment 1 | Controls | Ctrl1 | 89 | N/A | 150.403 | 14449.417 | 45316.823 | 4318.932 | 13.133 |
|  |  | Ctrl2 | 38.2 | N/A | 320.078 | 22002.270 | 78832.863 | 6637.652 | 17.265 |
|  |  | Ctrl3 | 81 | N/A | 104.805 | 9986.581 | 27667.951 | 2336.836 | 9.100 |
|  | PSEN1 mutations | INT4DEL | 52 | 38 | 73.442 | 7315.727 | 26359.376 | 5095.538 | 14.270 |
|  |  | Y115H | 51 | 35 | 84.403 | 7928.374 | 23963.329 | 6473.258 | 23.273 |
|  |  | M139V | 42 | 40 | 178.792 | 9924.062 | 47553.106 | 8039.160 | 17.875 |
|  |  | M146I | 48 | 48 | 92.273 | 8576.782 | 35137.173 | 5364.140 | 13.240 |
|  |  | R278I | 48 | 51 | 119.701 | 9637.302 | 35361.985 | 3864.934 | 51.140 |
|  |  | E280G.A | I2 | 42 | 12.195 | 11931.671 | 12246.976 | 1415.818 | 17.210 |
|  |  | E280G.B | I1 | 42 | 53.766 | 10836.685 | 24575.873 | 3486.186 | 53.178 |
|  |  | E184D | 81 | 40 | 121.727 | 11767.123 | 43107.532 | 8627.507 | 25.980 |
|  |  | P436S.A | 89 | 47 | 129.792 | 15321.013 | 48146.878 | 6869.341 | 62.238 |
|  |  | P436S.B | 89 | 47 | 133.649 | 16543.806 | 50009.540 | 7206.799 | 70.020 |
| Experiment 2 | Controls | Ctrl1 | 90 |  | 147.818 | 10045.151 | 36977.219 | 3544.345 | 12.373 |
|  |  | Ctrl2 | 52 |  | 119.909 | 14620.695 | 33318.734 | 2908.702 | 10.423 |
|  |  | Ctrl3 | 82 |  | 115.922 | 12349.783 | 29719.750 | 2887.840 | 9.598 |
|  | PSEN1 mutations | INT4DEL | 51 |  | 92.597 | 10703.796 | 34061.094 | 6546.007 | 17.825 |
|  |  | Y115H | 52 |  | 47.727 | 9616.769 | 14543.425 | 3208.927 | 10.975 |
|  |  | M139V | 43 |  | 113.338 | 9779.370 | 31112.232 | 5669.195 | 14.160 |
|  |  | M146I | 52 |  | 79.610 | 10607.469 | 32827.806 | 4883.806 | 11.480 |
|  |  | R278I | 52 |  | 45.338 | 7906.070 | 14142.485 | 1371.496 | 41.490 |
|  |  | E280G.A | IJ |  | 14.597 | 8236.618 | 11742.366 | 1780.091 | 22.728 |
|  |  | E280G.B | I2 |  | 98.584 | 14071.245 | 46169.204 | 6666.626 | 86.815 |
|  |  | E184D | 82 |  | 52.195 | 9945.624 | 19207.148 | 3942.657 | 10.665 |
|  |  | P436S.A | 90 |  | 84.416 | 14443.522 | 33347.868 | 5536.087 | 52.290 |
|  |  | P436S.B | 90 |  | 115.403 | 16875.052 | 45631.051 | 6962.605 | 64.570 |
| Experiment 3 | Controls | Ctrl1 | 91 |  | 119.649 | 12469.027 | 35184.736 | 3214.150 | 10.050 |
|  |  | Ctrl2 | 83 |  | 164.844 | 14690.457 | 43230.598 | 3812.693 | 11.823 |
|  | PSEN1 mutations | INT4DEL | 38.1 |  | 209.156 | 15778.436 | 81876.097 | 14564.249 | 32.655 |
|  |  | Y115H | 77 |  | 102.610 | 12170.507 | 30747.157 | 7029.599 | 19.098 |
|  |  | M139V | 51 |  | 86.169 | 12185.516 | 25185.745 | 4931.490 | 9.305 |
|  |  | M146I | 77 |  | 126.091 | 17367.935 | 54040.088 | 8450.398 | 17.070 |
|  |  | R278I | 77 |  | 179.065 | 15153.123 | 54500.780 | 5720.892 | 74.113 |
|  |  | E280G.A | 77 |  | 34.442 | 11483.827 | 22702.268 | 2941.243 | 35.058 |
|  |  | E280G.B | 77 |  | 65.169 | 12340.798 | 37773.888 | 5698.266 | 69.708 |
|  |  | E184D | 83 |  | 139.455 | 15676.796 | 52093.984 | 9623.856 | 28.348 |
|  |  | P436S.A | 91 |  | 114.831 | 17255.251 | 41613.515 | 6155.258 | 62.128 |
|  |  | P436S.B | 91 |  | 118.909 | 19176.322 | 44784.474 | 7057.368 | 61.183 |

**Supplementary Table 4. Previously reported mass spec data**

|  |  | Ab37 | Ab38 | Ab39 | Ab40 | Ab42 |
| --- | --- | --- | --- | --- | --- | --- |
| Ctrl2 | Sample 1 | 3.51 | 7.93 | 2.56 | 25.86 | 1.87 |
|  | Sample 2 | 3.82 | 6.73 | 2.54 | 22.53 | 2.09 |
|  |  |  |  |  |  |  |
| Ctrl3 | Sample 1 | 4.36 | 8.75 | 2.77 | 22.69 | 1.58 |
|  | Sample 2 | 5.46 | 8.89 | 4.63 | 24.33 | 3.14 |
|  |  |  |  |  |  |  |
| PSEN1 int4del | Sample 1 | 2.23 | 3.36 | 0.00 | 5.16 | 0.00 |
|  | Sample 2 | 2.90 | 5.84 | 2.99 | 23.34 | 3.08 |
|  | Sample 3 | 2.95 | 5.00 | 2.48 | 20.30 | 3.14 |
|  | Sample 4 | 2.78 | 5.27 | 2.64 | 25.45 | 3.51 |
|  |  |  |  |  |  |  |
| PSEN1 Y115H | Sample 1 | 0.00 | 7.01 | 0.00 | 14.54 | 0.00 |
|  | Sample 2 | 4.90 | 7.13 | 4.52 | 22.27 | 4.05 |
|  |  |  |  |  |  |  |
| PSEN1 M139V | Sample 1 | 3.47 | 6.11 | 3.71 | 25.43 | 3.46 |
|  | Sample 2 | 3.72 | 6.75 | 3.68 | 24.23 | 3.47 |
|  |  |  |  |  |  |  |
| PSEN1 M146I | Sample 1 | 2.92 | 7.20 | 2.21 | 18.90 | 3.13 |
|  | Sample 2 | 3.27 | 8.88 | 3.19 | 27.11 | 4.09 |
|  |  |  |  |  |  |  |
| PSEN1 R278I | Sample 1 | 4.83 | 8.20 | 3.14 | 24.53 | 2.13 |
|  | Sample 2 | 5.04 | 8.48 | 3.74 | 31.16 | 3.18 |

**Supplementary Materials Reference List**

1. Arber C, Toombs J, Lovejoy C, et al. Familial Alzheimer’s disease patient-derived neurons reveal distinct mutation-specific effects on amyloid beta. *Mol Psychiatry*. 2020;25(11):2919-2931. doi:10.1038/s41380-019-0410-8

2. Pantazis CB, Yang A, Lara E, et al. A reference human induced pluripotent stem cell line for large-scale collaborative studies. *Cell Stem Cell*. 2022;29(12):1685-1702.e22. doi:10.1016/j.stem.2022.11.004

3. Willumsen N, Arber C, Lovejoy C, et al. The PSEN1 E280G mutation leads to increased amyloid-β43 production in induced pluripotent stem cell neurons and deposition in brain tissue. *Brain Commun*. 2023;5(1). doi:10.1093/braincomms/fcac321

4. Arber C, Belder CRS, Tomczuk F, et al. The presenilin 1 mutation P436S causes familial Alzheimer’s disease with elevated Aβ43 and atypical clinical manifestations. *Alzheimer’s & Dementia*. 2024;20(7):4717-4726. doi:10.1002/alz.13904
